# *Brain Communications* early career researcher paper prize

**DOI:** 10.1093/braincomms/fcac328

**Published:** 2023-01-11

**Authors:** Tara L Spires-Jones

**Affiliations:** Edinburgh, UK

## Abstract

Our editor introduces an early career researcher prize for the first author of a paper published in Brain Communications in 2022.

## Graphical Abstract

**Graphical abstract:**
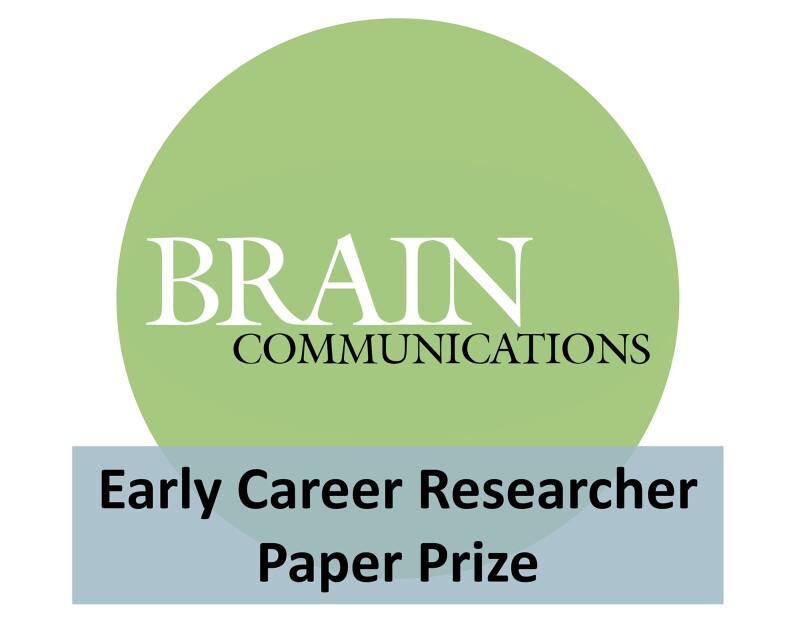

Welcome to Volume 5, issue 1 of *Brain Communications*. In this Editorial, I’m proud to announce that we will award a prize to the first author of one of the papers published in our journal in 2022. The aim of the prize is to recognize contributions of an early career researcher (student or postdoc) for their excellent translational neuroscience research. The winner will be invited to give a short talk about their paper at the annual Brain Journal conference online in March 2023. There will also be a small award for the winner, a nice addition of the prize to their CV, and of course bragging rights.

In order to make this prize happen, we need your help, dear readers! Please nominate a student or postdoc who is first author of one of our papers published in *Brain Communications* in 2022 by emailing brcoms.editorialoffice@oup.com. Nominations close at the end of January. We will ask the Editorial Board to vote on nominees, and the winner will be announced in February.

At *Brain Communications*, one of our goals is to foster career development of neuroscientists and we hope this prize will benefit the winner and more broadly highlight the outstanding contributions of early career researchers to our journal and to the field.

In addition to the new early career researcher paper prize, we have been keeping busy in the *Brain Comms* team. Recently I reported to the Guarantors of Brain, the charity that receives all profits from our journal, that *Brain Communications* is growing and exceeding performance predictions from our publisher Oxford University Press. Our editorial team was delighted to meet PhD students, clinicians, and postdoctoral researchers supported by fellowships made possible through our article processing fees at the annual Guarantors of Brain dinner.

The cover image for this issue comes from Mesbah *et al*.^1^ and shows the use of high-resolution MRI scans of the spinal cord as a tool to predict the optimum placement for the implantation of epidural stimulation, providing maximal coverage of the lumbosacral spinal cord to improve the functional outcomes of epidural stimulation in spinal cord injury population.
